# Resources Alter the Structure and Increase Stochasticity in Bromeliad Microfauna Communities

**DOI:** 10.1371/journal.pone.0118952

**Published:** 2015-03-16

**Authors:** Jana S. Petermann, Pavel Kratina, Nicholas A. C. Marino, A. Andrew M. MacDonald, Diane S. Srivastava

**Affiliations:** 1 Institute of Biology, Freie Universität Berlin, Königin-Luise-Str. 1–3, D-14195, Berlin, Germany; 2 Berlin-Brandenburg Institute of Advanced Biodiversity Research (BBIB), D-14195, Berlin, Germany; 3 Department of Ecology and Evolution, University of Salzburg, Hellbrunnerstrasse 34, 5020, Salzburg, Austria; 4 School of Biological and Chemical Sciences, Queen Mary University of London, London, E1 4NS, United Kingdom; 5 Department of Ecology, Biology Institute, Federal University of Rio de Janeiro (UFRJ), 7 Ilha do Fundão, Rio de Janeiro, RJ, PO Box 68020, Brazil; 6 Department of Zoology & Biodiversity Research Centre, University of British Columbia, 6270 University Blvd., Vancouver, BC, V6T 1Z4, Canada; Towson University, UNITED STATES

## Abstract

Although stochastic and deterministic processes have been found to jointly shape structure of natural communities, the relative importance of both forces may vary across different environmental conditions and across levels of biological organization. We tested the effects of abiotic environmental conditions, altered trophic interactions and dispersal limitation on the structure of aquatic microfauna communities in Costa Rican tank bromeliads. Our approach combined natural gradients in environmental conditions with experimental manipulations of bottom-up interactions (resources), top-down interactions (predators) and dispersal at two spatial scales in the field. We found that resource addition strongly increased the abundance and reduced the richness of microfauna communities. Community composition shifted in a predictable way towards assemblages dominated by flagellates and ciliates but with lower abundance and richness of algae and amoebae. While all functional groups responded strongly and predictably to resource addition, similarity among communities at the species level decreased, suggesting a role of stochasticity in species-level assembly processes. Dispersal limitation did not affect the communities. Since our design excluded potential priority effects we can attribute the differences in community similarity to increased demographic stochasticity of resource-enriched communities related to erratic changes in population sizes of some species. In contrast to resources, predators and environmental conditions had negligible effects on community structure. Our results demonstrate that bromeliad microfauna communities are strongly controlled by bottom-up forces. They further suggest that the relative importance of stochasticity may change with productivity and with the organizational level at which communities are examined.

## Introduction

Ecologists have long sought to understand which factors generate the variation within and among communities that is so ubiquitous in nature. Deterministic, niche-related processes such as environmental filtering [1], competition for resources [2] and top-down control [3] have been studied extensively. Species sorting resulting from these environmental conditions and species interactions can cause the predictable dominance of some taxa and the exclusion of others, causing communities of similar environments to converge in to similar species compositions. However, neutral forces such as demographic stochasticity can also generate structure within ecological communities via ecological drift [4]. Dispersal limitation is another structuring force that is predominantly regarded as neutral, even though dispersal rates can vary between organisms [5]. Deterministic and stochastic forces likely influence ecological communities in concert [6]. For example, species sorting resulting from interactions such as competition for resources and predation can be disrupted by dispersal [7], reducing variability among communities (i.e. lower beta diversity) but enhancing local richness by allowing excluded or novel species to enter the community [8–10].

Ecologists have yet to determine under which conditions, in which environments or for which organism groups particular structuring forces or their combinations prevail [11–13]. Partitioning the individual mechanisms in natural settings is particularly challenging as the interplay among these key forces often has complex effects on both local richness and compositional similarity among communities. Furthermore, priority events in the history of community assembly can mask potential deterministic influences on community structure [14].

In this study we use a natural system that can be relatively easily manipulated to conduct field tests of three deterministic structuring forces (environmental filtering, resource competition and predation) and one stochastic force (dispersal) identified as potentially important by more controlled but simplified systems [15]. Tank bromeliads are neotropical plants that harbor diverse multitrophic aquatic communities in the small water bodies that accumulate between their leaves (phytotelmata). These communities largely depend on leaf litter as a basal resource [16]. Due to their small size and relative autonomy from the surroundings bromeliad communities are useful systems for experimental studies involving *in situ* manipulations of community assembly. We focus on the microfauna communities in bromeliads, including all non-bacterial, non-viral microscopic organisms such as flagellates, ciliates, amoebae, algae and predatory microscopic metazoans such as rotifers and copepods. In nature, these communities are subject to varying environmental conditions within and among bromeliads such as water temperature and light regimes. Furthermore, resource input may vary strongly and the abundance of organisms at higher trophic levels may differ among bromeliads, resulting in differential strengths of bottom-up and top-down control on the microfauna. Dispersal is assumed to act as a neutral driver of community assembly since microfauna are mainly wind-, water- and vector-dispersed [17] and differences in dispersal abilities among species within these communities are relatively small. Because of the growth pattern of bromeliads their inhabiting communities are likely connected by hierarchically structured dispersal: local dispersal among the semi-independent leaf axils within a bromeliad likely may occur more frequently than regional dispersal between individual bromeliad plants.

Here, we examine the influence of environmental conditions, resources, predation and dispersal on bromeliad microfauna community structure. After homogenizing natural communities to exclude potential priority effects, we measured environmental variables and then explicitly manipulated resource input and predator presence in a field experiment. Because a spatial signal in community structure can result from effects of spatially correlated environmental variables as well as from dispersal limitation, we experimentally manipulated dispersal at local (among leaf axils) and regional spatial scales (among bromeliads). We then assessed effects of our experimental treatments by examining changes in the structure of microfauna communities consisting of five major functional groups (algae, amoebae, flagellates, ciliates and predatory microfauna).

We hypothesized that:

1. If deterministic processes dominate, then community structure will only respond to differences in environmental conditions and to the manipulation of species interactions such as resource competition and predation.
2. If dispersal as a stochastic process dominates, then community structure will change as a response to the experimental manipulation of dispersal at both, local and regional scales.
3. If deterministic processes and dispersal both contribute to community structure, then the experimental increase in regional dispersal rates (among bromeliads) should result in a much greater change in community composition than an experimental increase in local dispersal rates (within bromeliads). This is because distant sites will develop more different communities, through differences in environmental factors and species interactions, than communities within the same region, exposed to more similar conditions.

## Materials and Methods

### Study site and system

This study was carried out in April and May 2010 at the Estación Biológica Pitilla (10°59'N, 85°26'W) in the Área de Conservación Guanacaste (http://www.acguanacaste.ac.cr), north-western Costa Rica under research permit N° ACG-PI-028-2010 (Ministerio del Ambiente, Energía y Telecomunicaciones). The station is situated at an altitude of 700m and is surrounded by primary and secondary tropical rainforest and horse pastures. Bromeliads occur in all of these habitats. We used three dominant tank bromeliad species: *Werauhia sanguinolenta* (Linden ex Cogn. & Marchal) J.R. Grant, *W*. *gladioliflora* (H. Wendland) J.R. Grant and *W*. *kupperiana* (Suessenguth) J.R. Grant and initiated the experiment after the first strong rainfall of the wet season.

### Experimental set up and data collection

We selected similar-sized large bromeliads (diameter range: 0.98–2.15m, mean ± standard error of the mean (sem): 1.37±0.05m), in three habitats varying in canopy openness in order to include a large range in environmental conditions: secondary forest (relatively closed canopy with low light conditions), mixed habitat (mostly edge of the forest with mixed light conditions) and pasture (mostly open canopy with high light availability). We mixed water collected from 44 bromeliads growing in the same three habitats near our experimental bromeliads to include the same species of inhabiting organisms but homogenize the initial composition of experimental communities. We used a suction device with a tube to extract the water from the bromeliads after careful mixing to make sure all organisms were suspended in the water. With a filter with a mesh size of 850μm we removed large detritus particles and macroscopic organisms such as insect larvae (we checked for insect larvae emerging from eggs throughout the experiment and removed them instantly). Then, we added 35ml of this water, containing a mix of algae, protozoans and microscopic metazoans (hereafter collectively referred to as microfauna), bacteria, fungi and small detritus particles, to 50-ml falcon tubes. We placed 243 tubes containing the experimental communities into 27 bromeliads: nine bromeliads in each of the three habitats, nine tubes per bromeliad (for a schematic depiction of the experimental design see Supporting Information, Fig. A in S1 Supporting Information). Those 27 bromeliads were a subset of the 44 bromeliads from which the initial pool of species for the communities were collected.

The 27 experimental bromeliads were divided into three “trophic” treatments: control, increase of bottom-up effects by resource addition, addition of top-down effects by predator addition. For the resource-addition treatment, we collected leaf litter from the forest floor, chopped the material into 1-cm^2^ pieces and sterilized it by boiling it for two 10 minute intervals – with a two-day period in between to eliminate microfauna emerging from resting stages. We added 0.7g dry weight of sterilized litter to each experimental community in the resource-addition treatment group. This amount represents more than double the amount of litter we found on average in natural bromeliad tanks (Kratina et al. unpublished data). The experimental communities in the predator-addition treatment each received one large (late developmental stage, 4^th^ instar) and two small (early developmental stage, 2^nd^ instar) *Culex jenningsi* Dyar&Knab mosquito larvae. Those numbers represent average natural densities in bromeliads at the field site. Control communities did not receive any additions. All tubes were then covered with fine mosquito mesh to prevent insects from ovipositing and to monitor emerging adult mosquitoes. To keep predation pressure relatively constant over the course of the study, mosquito larvae were replaced whenever they pupated and emerged as adults. We could not observe the number of larvae during the study without disturbing the community, so we could not replace larvae that had died. However, this number was relatively low and all tubes in the predator treatment still contained mosquito larvae at the end of our study. The tubes were placed into separate leaf axils in the bromeliad with no water exchange between the tube and the bromeliad.

We nested a hierarchical dispersal manipulation within each trophic treatment in each bromeliad. In each bromeliad, three of the nine experimental communities served as manipulations of “regional dispersal” between bromeliads, i.e., the removal of any dispersal limitation at the regional scale. After gently mixing the contents of the tube, we extracted 1 ml of water from each of the three experimental communities in each of the three bromeliads of the same trophic treatment in the same habitat. We then mixed the resultant 9 ml in a common vial and redistributed 1 ml of this mixture into each regional dispersal community. Manipulations of “local dispersal” within bromeliads (i.e., the removal of dispersal limitation at the local scale) were treated similarly but only the 3 ml from the three “local” communities within a bromeliad were mixed. In three no-dispersal “control” communities we pipetted 1ml out of each community and placed the same liquid back into the tube to control for disturbance from pipetting. In these control communities, natural dispersal was the only means of exchange between communities, so patterns of dispersal limitation were allowed to develop over the course of the experiment. We performed the dispersal manipulations every four days over the four-week experimental duration.

At the start of the experiment, we measured the key structural variables that we expected to influence microfauna communities. These were bromeliad diameter, number of live leaves (as a measure of habitat complexity and of light availability in each leaf compartment), height above the ground, natural water content of the bromeliad (estimated visually in four classes from “dry” to “full”) and natural water content of each experimental leaf axil (in ml). Canopy openness was estimated by taking a photograph of the canopy above each bromeliad plant using a 35-mm lens and calculating the proportion of sky by pixel count. At the end of our study after four weeks, we measured pH, water temperature, volume, dissolved oxygen and turbidity in each tube to further describe the environment that the microfauna communities were experiencing. However, since these parameters can change across time and even within a day, their measurement at the end of the study only represents a snapshot of the environmental conditions and only large effects will be picked up by the analysis. We then collected microfauna samples from all 243 experimental communities and fixed them with acid 5%-iodine Lugol’s solution. The fixed samples were transferred to the University of British Columbia (Vancouver, Canada) where all organisms were counted under an inverted microscope (in 50-μl subsamples and 200x magnification). We used and expanded a photographic identification key developed for the microfauna living in bromeliads at our field site [18] to identify taxa based on their morphology and grouped them into “morphospecies” (hereafter referred to as “species”). The entire samples were screened for larger organisms (rotifers, copepods, flatworms, nematodes and mosquito larvae) under a dissecting scope. Species that occurred in extremely high numbers (several thousand per subsample) were counted in 20 fields of view (200x magnification) and their abundances per 50 μl estimated by extrapolation. All algal chain colonies were grouped into a single morphospecies and counted on a per-strand basis (multiple cells). Their abundance was calculated as the number of individual cells to be comparable with the other protozoan species using an average number of 17.65 cells per strand (based on a sample of n = 20, standard error of the mean = 5.11).

### Statistical analyses

#### Abundance and richness

We analyzed the effects of resource addition, predation and dispersal on the overall abundance and species richness (hereafter richness, our measure of alpha diversity) and on the abundance and richness of the main functional groups. All abundance data were natural-log-transformed to achieve normality and homogeneity of variance. This conservative approach removes samples with zero abundance from the analysis. We repeated all analyses after adding a value smaller than the smallest recorded density to the data before log transformation. These additional analyses showed the same results and are therefore not presented.

To account for the hierarchical error structure we analyzed abundance and richness with mixed effects models using the function lme in package *nlme* [19] in R, version 2.15.1 [20]. These analyses are described in more detail in the Supporting Information. To explore to what degree patterns in richness were simply due to variations in abundance, we used the function rarefy in R package *vegan* [21]. We additionally fit the mixed effects models with a number of different spatial autocorrelation functions [22]. Spatial autocorrelation did not show any effects (for details see Methods and Table A in S1 Supporting Information), so we present results from models without autocorrelation structure.

#### Community composition

We used multivariate analyses to test for the effects of canopy openness, temperature, trophic treatment and dispersal on community composition [23]. Our response variable was a Bray-Curtis-dissimilarity matrix resulting from a Hellinger-transformed species-by-site matrix [24], with “site” being one experimental community. We accounted for effects of spatial arrangement of the communities (see Supporting Information) and then conducted a permutational multivariate analysis of variance (PERMANOVA). After detecting significant trophic treatment effects on community composition, we examined dispersion of community composition within trophic treatment levels (i.e. how much the communities of one treatment differed among each other in terms of their composition). Differences in dispersion between levels of the dispersal manipulation were then analyzed using the same procedure.

To explore effects of varying alpha diversity (richness) on differences in community composition [11,25,26] we additionally used a Raup-Crick dissimilarity measure (see Supporting Information).

## Results

### Abundance and richness

We counted a total of 62 species of protozoa and micrometazoa in our experimental communities and classified them into five major functional groups: algae (11 species), flagellates (17 species), ciliates (11 species), amoebae (11 species), predatory microfauna (11 species, including rotifers, copepods and nematodes).

**Fig 1:**
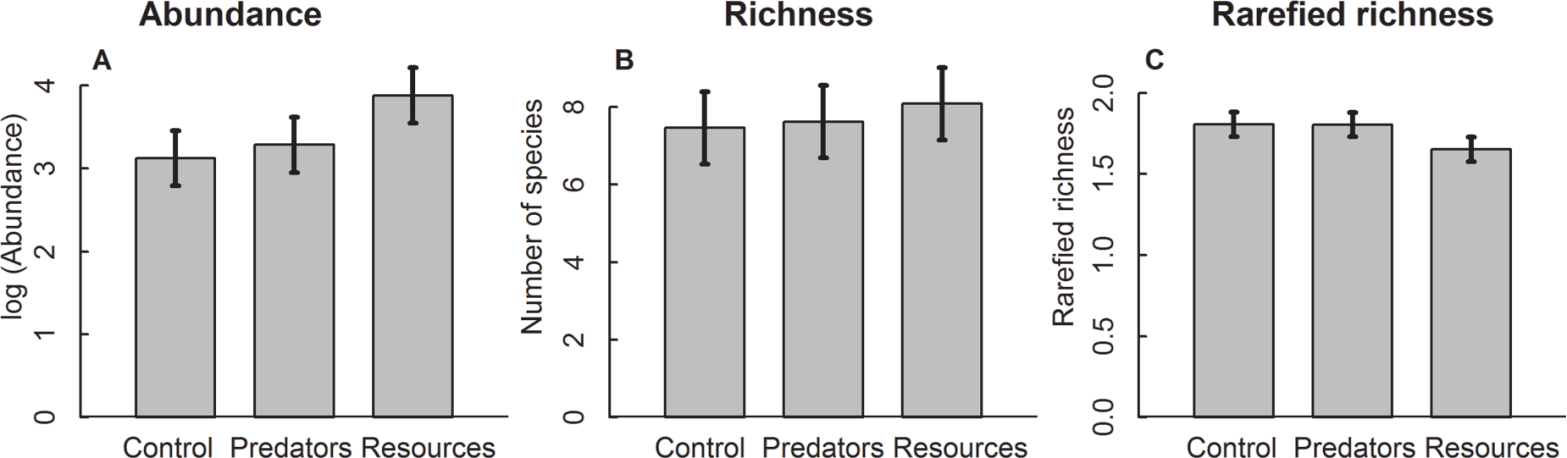
Mean log-transformed abundance (A), richness (B) and rarefied richness (C) of all microfauna for the three trophic treatment levels. Those levels are control, predator addition and resource addition. The numbers refer to a 50-μl subsample. Error bars show ± 1 standard error of the mean.

Overall, our trophic treatments had strong effects on microfauna abundance (Table 1), but largely due to the additions of resource rather than predators (Fig. 1A). Trophic treatment effects on overall richness only became apparent after data were rarefied because the large differences in abundance masked differences in richness in terms of the number of species per individuals. Rarefied richness in the resource-addition treatment was significantly lower compared with the other treatments (Table 1, Fig. 1B and C). Predator addition had no effect on overall microfauna abundance or richness (Fig. 1).

**Table 1:** Results of mixed effects model analyses for log-transformed abundance, richness and rarefied richness of all organisms combined, with “bromeliad” treated as a random effect.

			**Abundance**		**Richness**		**Rarefied richness**	
	**ndf**	**ddf**	**F**	**P**	**F**	**P**	**F**	**P**
**Sorter**	1	206	19.766	**<0.001**	38.532	**<0.001**	2.942	0.088
**Canopy openness**	1	23	4.483	**0.045**	1.511	0.231	2.685	0.115
**Trophic treatment** ^+^	2	23	10.572	**0.001**	0.757	0.48	5.953	**0.008**
**Temperature**	1	206	1.098	0.296	7.656	**0.006**	13.448	**<0.001**
**Dispersal** ^++^	2	206	2.191	0.114	0.798	0.452	1.699	0.185

^+^Trophic treatment levels: control, predator addition and resource addition.

^++^Dispersal levels: control, local dispersal and regional dispersal.

P-values<0.05 are printed in bold. ndf = numerator degrees of freedom, ddf = denumerator degrees of freedom.

**Fig 2:**
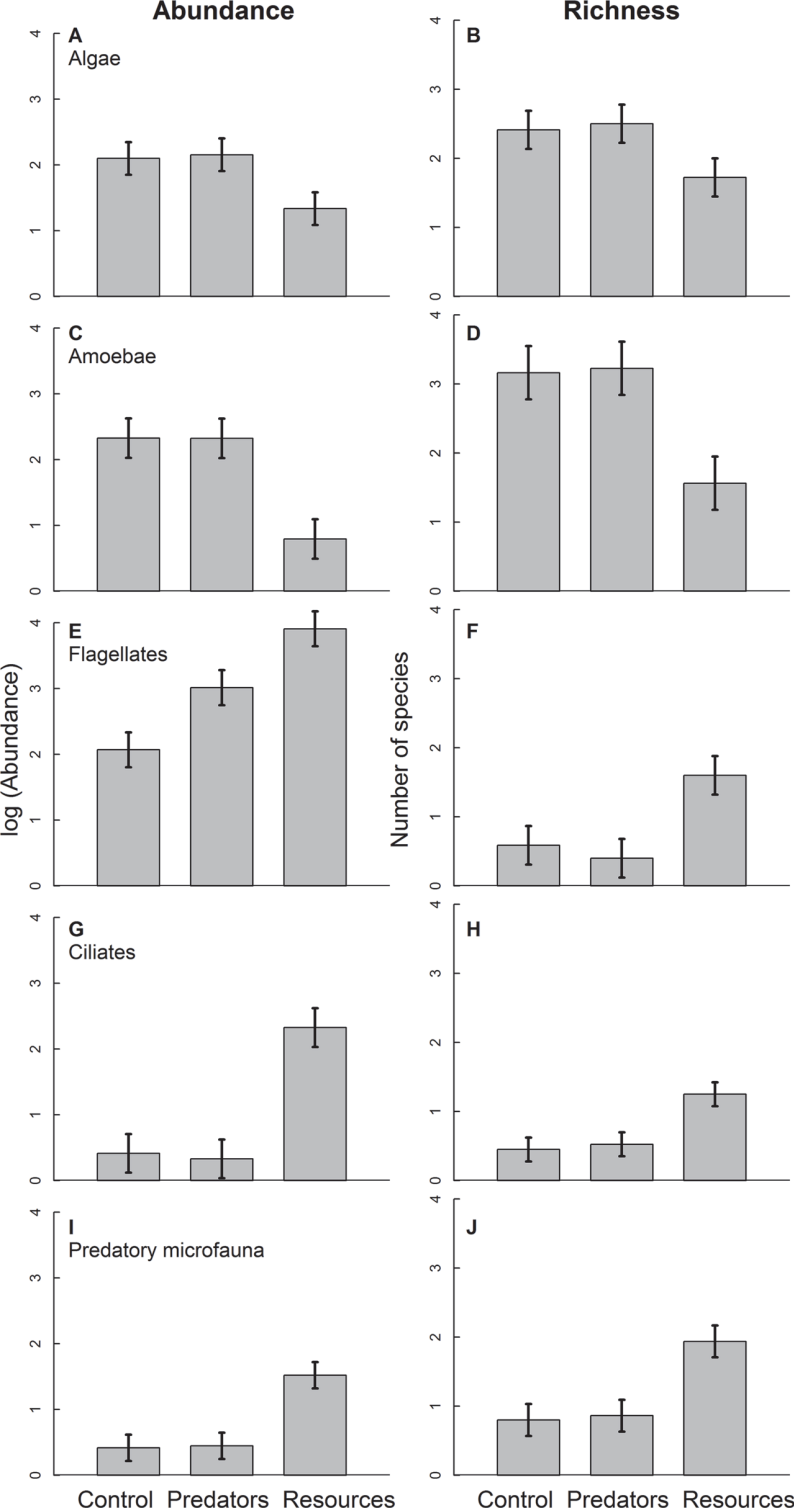
Mean log-transformed abundance and richness of algae (A, B), amoebae (C, D), flagellates (E, F), ciliates (G, H) and predatory microfauna (I, J). Those levels are control, predator addition and resource addition. The numbers refer to a 50-μl subsample. Error bars show ± 1 standard error of the mean.

Diverging patterns emerged for individual functional groups (Fig. 2, Tables 2 and 3). Abundance and richness of all groups responded to our trophic treatments, however, in different directions. This differential reaction shifted functional group composition. Algae and amoebae decreased in abundance and richness with resource addition (Fig. 2A, B, G, H) while the other groups increased (Fig. 2C, D, E, F, I, J). Flagellates were the only group that increased in abundance with predator addition (Fig. 2E). Trophic treatment effects on amoeba and ciliate richness disappeared after rarefication (F_2,23_ = 0.267, P = 0.768 and F_2,22_ = 1.520, P = 0.241, respectively), suggesting that differences in abundance were the main cause for the richness differences in these groups. Differences between trophic treatments in the other groups remained significant after rarefaction, indicating that changes in abundance did not completely explain effects of trophic treatments on the richness of algae, flagellates and predatory microfauna.

**Table 2:** Results of mixed effects model analyses for log-transformed abundance and richness of algae, amoebae and flagellates, with “bromeliad” treated as a random effect.

		**Algae: Abundance**			**Richness**		**Amoebae: Abundance**			**Richness**		**Flagellates: Abundance**			**Richness**	
	**ndf**	**ddf**	**F**	**P**	**F**	**P**	**ddf**	**F**	**P**	**F**	**P**	**ddf**	**F**	**P**	**F**	**P**
**Sorter**	1	189	46.736	**<0.001**	1.440	0.232	182	20.72	**<0.001**	14.127	**<0.001**	74	86.525	**<0.001**	42.291	**<0.001**
**Canopy openness**	1	23	0.041	0.841	3.521	0.073	23	0.774	0.388	1.750	0.199	19	0.304	0.588	0.09	0.768
**Trophic treatment** ^+^	2	23	25.644	**<0.001**	7.041	**0.004**	23	67.304	**<0.001**	23.208	**<0.001**	19	14.418	**<0.001**	7.369	**0.004**
**Temperature**	1	189	0.621	0.432	0.937	0.334	182	2.123	0.147	4.987	**0.027**	74	3.706	0.058	0.335	0.565
**Dispersal** ^++^	2	189	1.555	0.214	1.621	0.200	182	1.404	0.248	0.030	0.970	74	0.013	0.987	0.286	0.752

^+^Trophic treatment levels: control, predator addition and resource addition.

^++^Dispersal levels: control, local dispersal and regional dispersal, P-values<0.05 are printed in bold. ndf = numerator degrees of freedom, ddf = denumerator degrees of freedom

**Table 3:** Results of mixed effects model analyses for log-transformed abundance and richness of ciliates and predatory microfauna with “bromeliad” treated as a random effect.

		**Ciliates: Abundance**			**Richness**		**Predatory microfauna:Abundance**			**Richness**	
** **	**ndf**	**ddf**	**F**	**P**	**F**	**P**	**ddf**	**F**	**P**	**F**	**P**
**Sorter**	1	115	10.857	0.001	6.385	**0.013**	136	0.002	0.964	0.082	0.775
**Canopy openness**	1	22	2.412	0.135	1.422	0.246	23	0.365	0.552	0.132	0.720
**Trophic treatment** ^+^	2	22	87.189	**<0.001**	3.594	**0.045**	23	41.123	**<0.001**	20.206	**<0.001**
**Temperature**	1	115	5.461	0.021	0.645	0.423	136	0.680	0.411	0.450	0.504
**Dispersal** ^++^	2	115	0.733	0.483	2.692	0.072	136	0.001	0.999	0.455	0.635

^+^Trophic treatment levels: control, predator addition and resource addition.

^++^Dispersal levels: control, local dispersal and regional dispersal, P-values<0.05 are printed in bold. ndf = numerator degrees of freedom, ddf = denumerator degrees of freedom

Only two environmental variables had significant effects on the experimental communities: canopy openness and water temperature. The other environmental variables were removed from further analyses. Canopy openness and water temperature both showed a spatial signal (i.e., they were both spatially autocorrelated). Canopy openness influenced overall microfauna abundance (Table 1) with higher abundance in bromeliads under more open canopies. However, this effect was driven by three bromeliads with very open canopy conditions (>40% canopy openness) and was not significant when those were removed. Similarly, the negative effect of water temperature on overall microfauna richness (Table 1) was largely caused by two experimental communities with extremely high temperatures (>32°C) and very low richness (≤3 species). Negative effects of water temperatures on the abundance of ciliates and on the richness of amoebae (Tables 2 and 3), were also driven by these two communities.

The dispersal manipulation had no effect on abundance or richness of the combined microfaunal community, or of any of the functional groups (Tables 1 and 2). Interactions between the trophic treatment and the dispersal manipulation (and other interactions such as canopy openness x dispersal and temperature x dispersal) were also not significant in any analysis. Including spatial autocorrelation structures into the models never improved their fit (Supporting Information, Table A in S1 Supporting Information), indicating that the spatial arrangement of bromeliads was not an important driver of microfauna abundance or richness. Only models without autocorrelation structure and without interaction terms are therefore presented.

### Community composition

The spatial arrangement of the communities had an influence on their composition (F_2,234_ = 1.8099, P = 0.005 for linear effects, F_4,232_ = 2.8058, P = 0.005 for the four selected PCNM vectors) but this did not qualitatively affect any results. In the following section we present only results from analyses in which we removed the spatial trends.

**Fig 3:**
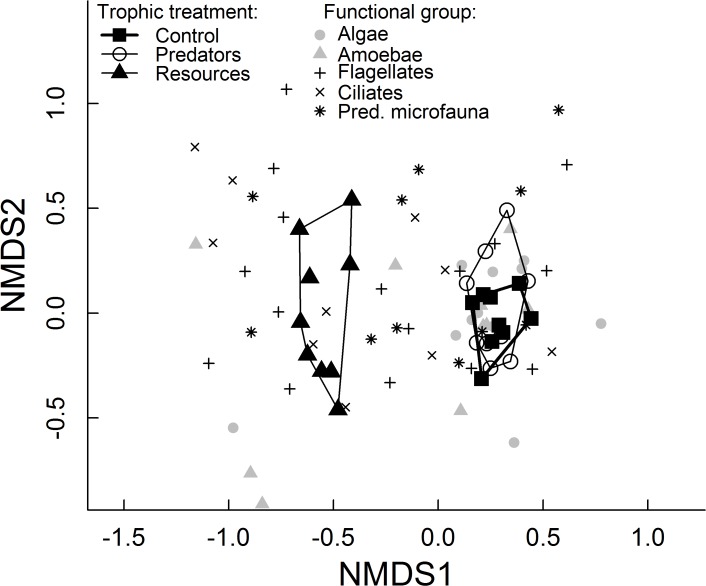
Non-metric multidimensional scaling (NMDS) representation of community- and species responses to nutrient and predator addition. Average microfauna communities are shown as large symbols with contours drawn around communities of the same trophic treatment (total n = 27). Species are depicted by smaller symbols coded according to their functional group identity. Stress = 0.10, non-metric fit R^2^ = 0.99, linear fit R^2^ = 0.95.

We found that experimental communities with additional resources were dramatically different in their species composition from control and predator-addition communities (Fig. 3, Table 4 and Supporting Information, Fig. B in S1 Supporting Information). Furthermore, dispersion of community composition (manifested in the spread of symbols of communities in Fig. 3 and measured as their distance to the centroid) among communities within the resource-addition treatment was larger than among communities within the control and predator-addition treatments, respectively (F_2,234_ = 3.267, P = 0.040). This means that communities receiving added resources were more dissimilar from each other (higher beta diversity) than control communities and communities with predators. Communities with different dispersal manipulations did not differ in their community composition (Table 4), nor did within-group dispersion differ between dispersal manipulations (F_2,234_ = 1.298, P = 0.275). We detected a small effect (see R^2^, Table 4) of canopy openness and temperature on microfauna species composition. Removing abundance differences between communities by rarefying the community matrix before the analysis did not change differences in composition but removed differences in dispersion (results not shown). This suggests that the differences in dispersion are largely driven by changes in population sizes of some species.

**Table 4:** Effects of environmental and experimental variables on community composition. Results are from a PERMANOVA on the Bray-Curtis dissimilarity matrix of residuals from the spatial model.

	**df**	**SS**	**MS**	**F**	**R^2^**	**P**
**Sorter**	1	0.642	0.642	13.866	0.048	**<0.001**
**Canopy openness**	1	0.295	0.295	6.382	0.022	**<0.001**
**Trophic treatment** ^+^	2	1.544	0.772	16.679	0.116	**<0.001**
**Temperature**	1	0.196	0.196	4.23	0.015	**<0.001**
**Dispersal** ^++^	2	0.05	0.025	0.537	0.004	0.984
**Residuals**	229	10.597	0.046		0.795	
**Total**	236	13.323			1	

^+^Trophic treatment levels were: control, predator addition and resource addition.

^++^Dispersal levels were: control, local dispersal, regional dispersal.

P-values<0.05 are printed in bold. ndf = numerator degrees of freedom, ddf = denumerator degrees of freedom.

Differences in composition between communities under different trophic treatments did not solely result from differences in alpha diversities since the analysis of the modified Raup-Crick dissimilarity matrix gave equivalent results to the ones from the Bray-Curtis matrix. However, canopy openness and temperature did not show significant effects on community composition when using the Raup-Crick metric (Supporting Information, Table B in S1 Supporting Information), so differences in composition related to those two environmental variables were likely driven by changes in alpha diversities.

## Discussion

The aim of this study was to disentangle the role of deterministic niche processes from the more stochastic process of dispersal limitation in structuring natural bromeliad microfauna communities. We found strong effects of detrital resources on community abundances, richness and composition, but little effect of dispersal limitation either alone or in combination with niche-based processes. These results substantially support our first hypothesis of a strong influence of species sorting. Resource addition resulted in predictable shifts in functional group composition but caused an increase in the dispersion of community composition at the species level, suggesting that the level of organization (functional group vs. species composition) may define the relative importance of deterministic and stochastic processes.

### Deterministic processes: bottom-up

Bottom-up control was the strongest and most prevalent effect driving several community characteristics, irrespective of differences in environmental conditions or experimental dispersal levels. Adding resources in the form of leaf litter increased overall microfauna abundance, decreased rarefied richness and shifted species composition. Abundances of certain fast-growing species were boosted strongly by the additional resources while overall species incidences remained unchanged, resulting in communities with less species per number of individuals. The decrease in alpha diversity with increased resources agrees with studies from grasslands [27] where strong competitors dominate the plant communities after fertilization.

In our study, resource addition also shifted functional group composition of microfauna communities. Flagellates (the most abundant group) increased most strongly as a response to resource addition, driving overall abundance patterns. Flagellates have short generation times [28] and have been found to respond rapidly to changing conditions [29]. These groups can exploit additional resources more quickly than other groups and therefore benefitted from nutrient addition most, at least in the short term [30]. Predatory microfauna (rotifers and copepods) likely increased in their abundance and richness as a response to the increasing abundance of their prey (flagellates and ciliates). In contrast to the other groups, algae and amoebae declined in abundance and richness with increased resources. The photoautotroph algae likely declined with the associated decrease in light caused by leaf litter addition. Amoebae are known to show relatively slow responses to changing environmental conditions [31] and were likely outcompeted by the fast-responding groups (flagellates and ciliates).

### Deterministic processes: top-down

Unexpectedly, only flagellate abundance increased with predator presence while overall microfauna abundance and the abundance of other groups did not change. Flagellates might have benefited from predators through a reduction in competition or predation by other groups possibly arising from size-selective feeding by mosquitoes [32], but more likely, nutrient cycling enhanced by predators (shown for higher trophic levels in bromeliads by [33]) might have benefited the flagellates disproportionally in a way similar to the resource treatment.

It is not clear why the other groups did not change in response to the predators. Although we mimicked average natural densities of mosquitoes in bromeliads in the area (Srivastava, unpublished data; [34,35]), our experimental densities were kept constant. Yet, mosquito densities vary greatly between leaf axils even within bromeliads (pers. obs.) due to oviposition events and to predation pressure from larvae of the damselfly *Mecistogaster modesta* Selys [36,37]. Moreover, the microfauna communities themselves can consist of several more or less distinct trophic levels, potentially preventing the emergence of clear, unidirectional effects. A number of studies have furthermore shown rapid plastic changes (e.g., the formation of resting stages) or evolutionary adaptations of protozoan and algal prey species to the presence of predators, resulting in a dampening of top-down control [38,39]. For these reasons, we remain cautious in the interpretation of the weak predator effects on microfauna communities.

Deterministic processes: environmental conditions

Only canopy openness and temperature had effects on community structure, and those were entirely due to high-temperature or high-canopy openness outlier communities. In a parallel observational study a number of habitat variables also influenced the structure of natural microfauna communities (canopy openness, height of the bromeliad above ground and for individual functional groups also pH, temperature and the number of live leaves of the bromeliad), however, explained variance was relatively low (Kratina et al. unpublished data).

The microfauna communities in Brazilian coastal bromeliads also show significant, but weak (17% of the variance) correlations with environmental factors, notably temperature, chlorophyll-*a*, water volume and water colour [40]. One potential explanation of the weak effects of environmental variables in our experiment is that the tubes buffered the organisms from the natural range in conditions. We found that pH and oxygen concentrations in the experiment were less variable and had different mean values (higher pH and lower oxygen concentrations) than in natural leaf wells.

### Stochastic process: dispersal limitation

While we detected a spatial signal in community composition, its inclusion in the models did not change the strong influence of the trophic treatment or the lack of dispersal effects. Furthermore, we did not find an influence of spatial arrangement on abundance and richness, and dispersal treatments had no effect on community structure. Instead, communities appeared to maintain their initial composition over the duration of the experiment, with most species being present at low abundance and extinctions being rare. We considered dispersal limitation a stochastic factor. However, had it affected only certain species, so operating in a deterministic fashion, we would similarly have seen changes in community composition following our dispersal manipulations.

Environmental differences between bromeliads in the field did not lead to strong species sorting in our experiment, although natural bromeliad communities differ from each other (Kratina et al. unpublished data). One interpretation of our results is that by homogenizing microfauna communities we overcame any priority effects of dispersal limitation that underlay these natural community differences. Whereas dispersal limitation has been detected in pitcher plant microfauna [41], and low prey dispersal can increase regional diversity in pond zooplankton [42], dispersal manipulation does not always affect local diversity [42,43]. Our results agree with other studies of bromeliad microfauna finding no relationship between spatial distance among bromeliads and community composition [40]. This suggests that dispersal is generally not limiting at these spatial scales (∼100 m) in bromeliad microfauna. However, since these organisms are rarely specialized on bromeliads as a habitat, other water bodies as source habitats should be taken into account in future studies, even if they only exist temporarily (such as puddles or water-filled flowers, [44]). Protozoa and small metazoa may be dispersed by wind, water or by other organisms that act as biotic vectors [17,45]. Dispersal via these vectors likely leads to relatively similar dispersal rates across species in microfauna communities in bromeliads, i.e. neutral dispersal processes. However, microfauna propagule characteristics can play a role in determining dispersal rate and distance as well, especially over small distances [5,45–47]. Unfortunately, our knowledge on dispersal processes in microorganisms in natural communities is still limited [48].

### The relative importance of deterministic versus stochastic forces

Our study of species-rich natural communities strengthens the results from more controlled experimental mesocosms [15] by showing a stronger influence of stochasticity on species composition under high resource input compared with control conditions. We established our experimental communities with non-diluted water from natural bromeliads containing protists at high natural densities with populations likely being close to their carrying capacity. The homogenization at the start of the experiment and the direct crossed dispersal treatment allowed us to exclude priority effects and dispersal limitation, leaving demographic stochasticity (i.e. stochastic mortality and reproduction) as the likely underlying mechanism producing dissimilarity over time among communities with added resources.

Certain species benefitted from resource addition more than other species but the identity of those dominant species varied among replicate communities. This increase in stochasticity with increased resource availability has been shown previously from grasslands, where beta diversity of plant communities was positively related to nitrogen enrichment [49] or site productivity [50], and from lakes, where higher energy availability increased the beta diversity of fish communities [51]. However, in another study, the alpha and beta diversity of lake benthic invertebrate communities was reduced after nutrient enrichment [52]. One of the underlying mechanisms that have been proposed to drive changes in beta diversity along the productivity gradient is cyclic species turnover which is expected to be faster under increased productivity [53]. Unfortunately, the detection of turnover processes requires detailed long-term data sets that are rarely available.

In contrast to the increased stochasticity at the species level with higher resource availability, our study shows that at the functional-group level, shifts in composition towards communities dominated by flagellates and ciliates were strong and predictable. The general pattern emerging from this and other studies is that not only are stochastic and deterministic forces jointly structuring communities [6] but we are beginning to identify particular conditions and levels of organization (i.e. species vs. functional groups) at which a given structuring force is relatively more important. At functionally defined higher-order levels that integrate over species (e.g., functional groups) community composition appears to be driven more strongly by deterministic forces such as competition, and is therefore relatively predictable. Below these levels, communities are subject to stochastic assembly processes and their species composition is less predictable. Similar results were found for temperate grassland communities whose functional group composition converged predictably over the course of community assembly [54]. In grasslands, the composition of species traits has also been found to converge in a deterministic fashion while the identity of coexisting species was more strongly driven by priority effects [55,56]. These patterns might ultimately result from self-organized assembly processes [57]. Whereas trait-based analyses have proven useful in community assembly and ecosystem function studies in other systems they have rarely been attempted with microfauna (but see [58,59,60]). These analyses could improve our understanding of the structure and dynamics of complex natural communities and remain a promising venue for future research.

## Supporting Information

S1 Supporting Information Supporting information including Supporting Statistical Methods, Fig. A, Fig. B, Table A and Table B. (DOCX)
