# Supplementary material for: Resources Alter the Structure and Increase Stochasticity in Bromeliad Microfauna Communities
Source: PLoS One. 2015 Mar 16;10(3):e0118952. doi: 10.1371/journal.pone.0118952 (PMC4361661; doi:10.1371/journal.pone.0118952)
Supplement: S1 Supporting Information — (DOCX) [file pone.0118952.s001.docx]

**Resources alter the structure and increase stochasticity in bromeliad microfauna communities**

**Jana S. Petermann^1,2,3,*^, Pavel Kratina^4^, Nicholas A. C. Marino^5^, A. Andrew M. MacDonald^6^ and Diane S. Srivastava^6^**

^1^Institute of Biology, Freie Universität Berlin, Königin-Luise-Str. 1-3, D-14195 Berlin, Germany
^2^Berlin-Brandenburg Institute of Advanced Biodiversity Research (BBIB), D-14195 Berlin, Germany
^3^Department of Ecology and Evolution, University of Salzburg, Hellbrunnerstrasse 34, 5020 Salzburg, Austria
^4^ School of Biological and Chemical Sciences, Queen Mary University of London, London E1 4NS, UK
^5^Department of Ecology, Biology Institute, Federal University of Rio de Janeiro (UFRJ), 7 Ilha do Fundão, Rio de Janeiro, RJ, PO Box 68020, Brazil
^6^Department of Zoology & Biodiversity Research Centre, University of British Columbia, 6270 University Blvd., Vancouver BC, V6T 1Z4, Canada
* Corresponding author: [jana.petermann@fu-berlin.de](mailto:jana.petermann@fu-berlin.de)

**Supporting Information**

**Statistical Methods**

*Abundance and richness*

*Mixed effects models*

Abundance and richness were analyzed with mixed effects models using the function lme in package *nlme* [[1](#_ENREF_1)] in R and treating "bromeliads" as a random effect. The identity of the person sorting the samples was included as a covariable "sorter" to remove additional variation from the models. The effects of all environmental variables were tested initially by including them as covariables in the models. The *a priori* models followed the structure:

y ~ sorter + environmental variables at larger spatial scale (e.g., canopy cover) + trophic treatment + interactions (environment:trophic treatment)

+ environmental variables at bromeliad scale (e.g., water temperature) + dispersal+ interactions (trophic treatment:dispersal)+ interactions (environment:dispersal),

random=~1|bromeliad

Non-significant environmental variables and interactions were dropped sequentially from the analyses during model selection procedures.

*Abundance-richness relationships*

We used the function rarefy in R package *vegan* [[2](#_ENREF_2)] in order to test whether patterns in richness were simply due to variations in abundance. This procedure randomly samples species for a fixed low number of individuals. We decided to rarefy to two individuals per subsample as this was the lowest abundance recorded in our study because). We then analyzed rarefied richness of the overall community and of individual functional groups the same way as original richness.

We furthermore repeated all analyses after excluding rare morphospecies, defined as having a total abundance in all samples ≤10 individuals (29 of the 62 morphospecies).

*Autocorrelation in mixed effects models*

We fit the mixed effects models testing abundance and richness with a number of different spatial autocorrelation functions [[3](#_ENREF_3)] using GPS coordinates of individual bromeliads. Since very little is known about the spatial distribution characteristics and dispersal of microfauna, we did not have an *a priori* expectation for the type of autocorrelation. The correlation functions we tested were: exponential, Gaussian, linear, rational quadratic and spherical. For the purpose of assigning spatial coordinates, the nine different communities within individual bromeliads were assumed to be arranged in a 20 x 20 cm grid with regular spacing of 10 cm. We compared models without and with different autocorrelation functions using the Akaike’s Information Criterion (AIC, [[4](#_ENREF_4)]). There was no significant difference in AIC values (Table A) among these models so we present results from models without autocorrelation structure.

*Community composition*

*Spatial effects on community composition*

In order to account for spatial effects potentially driven by natural dispersal during the experiment, we used Constrained Correspondence Analysis (CCA) with spatial coordinates to obtain linearly detrended residuals. If there were significant linear trends, these residuals were used in an analysis of principal coordinates of neighbour matrices [[PCNM, 5](#_ENREF_5)] using the library *spacemakeR.* In the absence of linear trends Hellinger-transformed community data were used in the PCNM without detrending. We selected PCNM vectors for the final spatial model based on the lowest AIC values with the function ortho.AIC in *spacemakeR*.

*Testing for effects of trophic treatment and dispersal limitation*

After fitting a CCA with the final spatial model, we used the adonis function in *vegan* to conduct a permutational multivariate analysis of variance (PERMANOVA) with 9999 permutations on the residuals of the community matrices.When significant trophic treatment effects community composition were detected, we used the function betadisper in *vegan* to examine dispersion of community composition within trophic treatment levels. Differences in dispersion were analyzed with the function permutest using 9999 permutations. Differences in dispersion between levels of the dispersal manipulation were then analyzed using similar methods. We visualized the results for species composition with non-metric multidimensional scaling (NMDS) using the metaMDS function in vegan (maximum of 20 random starts, three dimensions).

*Null model to check for effect of varying alpha diversity*

Differences in composition among communities (beta diversity) can result from differences in alpha or gamma diversities [[6-8](#_ENREF_6)]. We assume gamma diversity (regional pool size) to be constant across communities in our study. However, in order to compare our results to null expectations with the same alpha diversities, we also analyzed a modified Raup-Crick dissimilarity measure (non-standardized version) using an R script from Chase et al. [[6](#_ENREF_6)].

**Figures
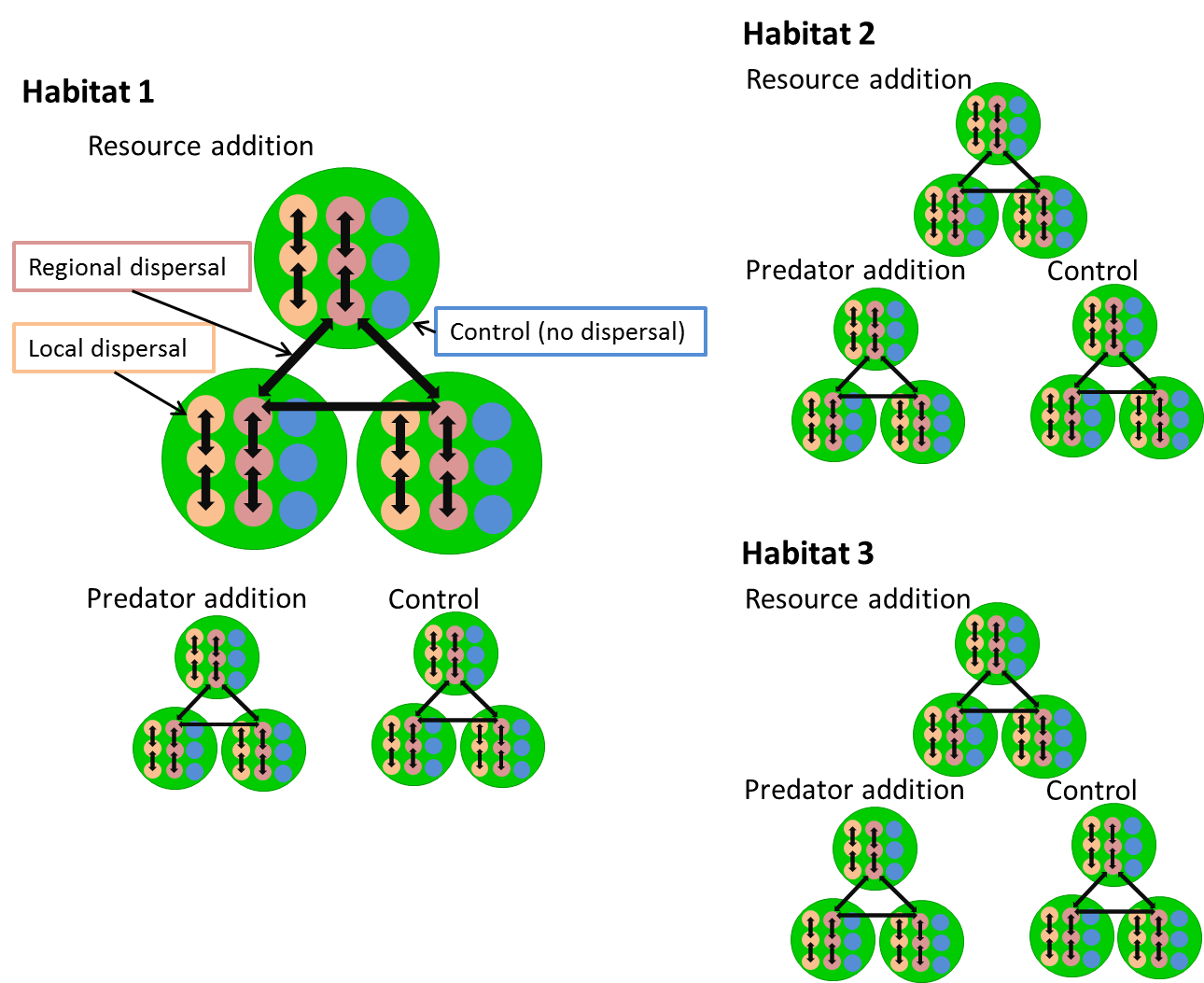
**

**Fig. A** Graphical depiction of the experimental design. The experiment was set up using the same design in three habitats (secondary forest, mixed habitat and pasture), i.e., as a block design, in order to include a large range in environmental conditions. In each habitat, three bromeliads (green circles) were haphazardly assigned to one trophic treatment (resource addition, predator addition or control). Within each bromeliad there were nine communities, randomly assigned to three different manipulations of dispersal limitation: control (no artificial dispersal, natural dispersal only), experimental (artificial) local dispersal and experimental (artificial) regional dispersal. Experimental local dispersal took place among the three communities of that treatment within each bromeliad. Experimental regional dispersal included experimental local dispersal but additional experimental dispersal among the “regional dispersal” communities of the two other bromeliads of the same trophic treatment in the same habitat (for detailed methods see main text).

**
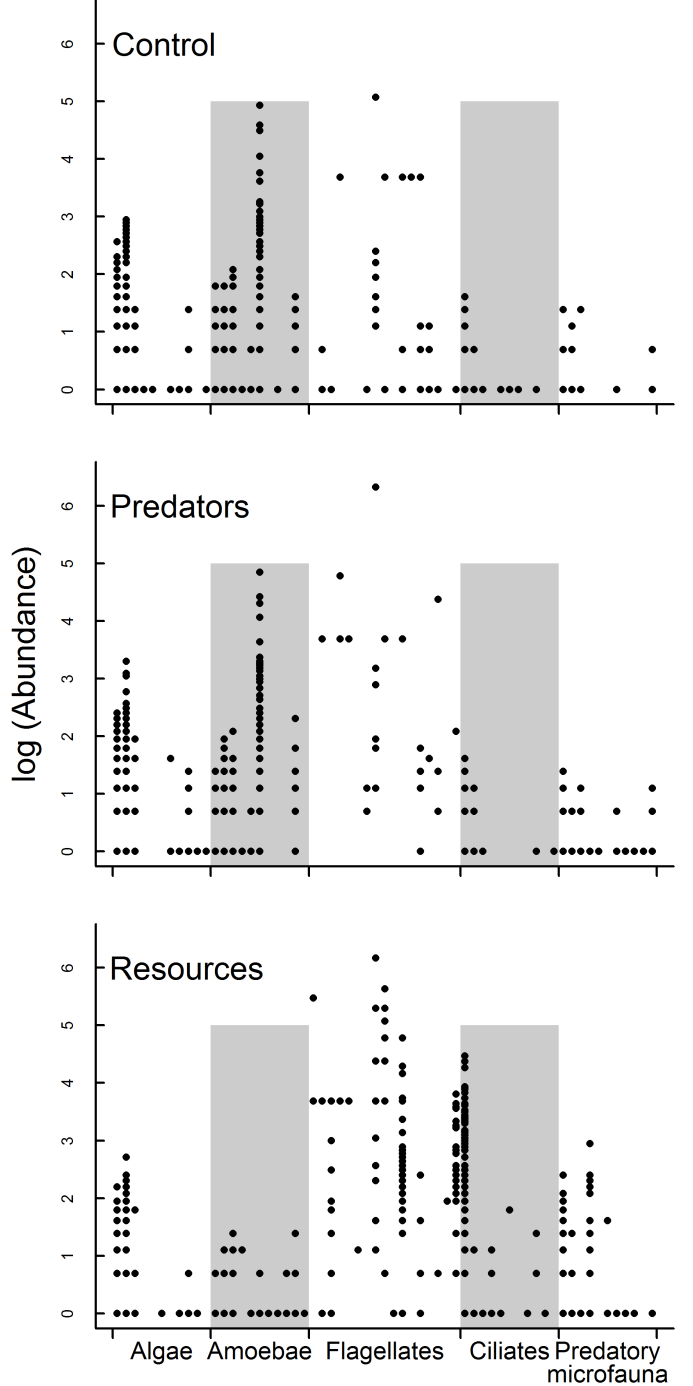

Fig. B** Log-transformed abundance of all 62 morphospecies in a 50-μl subsample from each experimental community binned into five major functional groups: algae, amoebae, flagellates, ciliates and predatory microfauna. Panels represent abundance in the three trophic treatment levels: control, predator addition and resource addition. The grey background is included for better visibility of functional group identities.

**Tables**

**Table A:** Comparison of candidate mixed effects models with different spatial autocorrelation classes fit with function lme in package *nlme.* Values of Akaike’s Information Criterion (AIC) are given for models with microfauna abundance (log transformed) and richness as response variables. The best model (lowest AIC) is highlighted in bold print. However, the model with the highest and lowest AIC are not significantly different from each other (P=0.0619 for abundance and P=0.8561 for richness, respectively).

| **corStruct class** | **Autocorrelation type** | **AIC abundance** | **AIC richness** |
| --- | --- | --- | --- |
| none | NA | 706.083 | **1184.686** |
| corExp | exponential spatial | 705.150 | 1186.620 |
| corGaus | Gaussian spatial | **704.599** | 1186.651 |
| corLin | linear spatial | 704.620 | 1186.653 |
| corRatio | rational quadratics spatial | 705.730 | 1186.556 |
| corSpher | spherical spatial | 704.620 | 1186.653 |

**Table B:** Effects of environmental and experimental variables on community composition. Results are from a PERMANOVA on a modified Raup-Crick measure of community dissimilarity of residuals from the final spatial model.

|  | **df** | | **SS** | **MS** | **F** | **R^2^** | **P** |
| --- | --- | --- | --- | --- | --- | --- | --- |
| **Sorter** | | 1 | 0.117 | 0.117 | 0.935 | 0.004 | 0.911 |
| **Canopy openness** | | 1 | 0.127 | 0.127 | 1.018 | 0.004 | 0.324 |
| **Trophic treatment** | | 2 | 0.259 | 0.13 | 1.036 | 0.009 | **0.027** |
| **Temperature** | | 1 | 0.123 | 0.123 | 0.98 | 0.004 | 0.801 |
| **Dispersal** | | 2 | 0.254 | 0.127 | 1.015 | 0.009 | 0.387 |
| **Residuals** | | 229 | 28.686 | 0.125 |  | 0.97 |  |
| **Total** | | 236 | 29.567 |  |  | 1 |  |

+Trophic treatment levels: control, predator addition and resource addition.
++Dispersal levels: control, local dispersal and regional dispersal.

P-values<0.05 are printed in bold. df=degrees of freedom, SS=Sum of Squares, MS=Mean Square.

**References**

1. Pinheiro JC, Bates DM, DebRoy S, Sarkar D, R Development Core Team. nlme: Linear and Nonlinear Mixed Effects Models. R package version 3.1-102; 2011.

2. Oksanen J, Blanchet FG, Kindt R, Legendre P, O'Hara B, Simpson GL, et al. vegan: Community Ecology Package. R package version 1.17-11; 2011.

3. Pinheiro JC, Bates DM. Mixed-Effects Models in S and S-PLUS. New York: Springer; 2000.

4. Burnham KP, Anderson DR. Model Selection and Multimodel Inference: A Practical Information-theoretic Approach. New York: Springer; 2002.

5. Borcard D, Legendre P, Avois-Jacquet C, Tuomisto H. Dissecting spatial structure of ecological data at multiple scales. Ecology 2004; 85: 1826-1832.

6. Chase JM, Kraft NJB, Smith KG, Vellend M, Inouye BD. Using null models to disentangle variation in community dissimilarity from variation in a-diversity. Ecosphere 2011; 2: 1-11.

7. Chase JM, Myers JA. Disentangling the importance of ecological niches from stochastic processes across scales. Philosophical Transactions of the Royal Society B: Biological Sciences 2011; 366: 2351-2363.

8. Kraft NJB, Comita LS, Chase JM, Sanders NJ, Swenson NG, Crist TO, et al. Disentangling the drivers of β diversity along latitudinal and elevational gradients. Science 2011; 333: 1755-1758.
